# Risk of Fall-Related Injuries Associated with Antidepressant Use in Elderly Patients: A Nationwide Matched Cohort Study

**DOI:** 10.3390/ijerph19042298

**Published:** 2022-02-17

**Authors:** Yu-Seon Jung, David Suh, Hang-Seok Choi, Hee-Deok Park, Sun-Young Jung, Dong-Churl Suh

**Affiliations:** 1 Department of Pharmacy, College of Pharmacy, Chung-Ang University, Seoul 06974, Korea; yuseon.jung72@gmail.com (Y.-S.J.); phd7813@pharmgenscience.com (H.-D.P.); 2 School of Public Health, University of Michigan, Ann Arbor, MI 48109, USA; sdivad87@gmail.com; 3 Department of Biostatistics, College of Medicine, Korea University, Seoul 02841, Korea; neuldol@gmail.com

**Keywords:** antidepressant, fall-related injuries, older adults, concurrent medications, polypharmacy

## Abstract

Previous studies have reported a higher risk of falls among tricyclic antidepressant (TCA) users compared to selective serotonin reuptake inhibitor (SSRI) users, yet SSRIs are known as a safer antidepressant class for use in older adults. This study examined the effects of antidepressant use on the risk of fall-related injuries after classifying antidepressant drugs, polypharmacy, and central nervous system (CNS) drugs by therapeutic classes and identifying factors influencing risk of fall-related injuries. A retrospective matched cohort study based on propensity scores was conducted among older adults, aged 70–89 years, who initiated antidepressant use between 1 January 2012 and 31 December 2014 using the national health insurance system senior cohort in Korea. The proportional hazard Cox regression model was used to examine the association between fall-related injuries and antidepressants. The subgroup analyses were performed to assess the risk of fall-related injuries by the number of concurrently administered medications, therapeutic classes of antidepressants, and CNS class medications. This study found that duloxetine, escitalopram, paroxetine, amitriptyline, imipramine, and trazodone significantly increased the risk of fall-related injuries in older adults. When antidepressants were prescribed to older adults, prescribers carefully considered factors including the dose, number of concurrently administered medications, and therapeutic classes of CNS.

## 1. Introduction

The choice of antidepressant in clinical practice is based on patient preferences and the safety profile of the drug [1,2]. Caution is particularly needed for older adults who are vulnerable to potential drug-related adverse events. Selective serotonin reuptake inhibitors (SSRIs) and serotonin norepinephrine reuptake inhibitors (SNRIs) are considered to be a relatively safe antidepressant choice and are recommended over tricyclic antidepressants (TCAs) for older adults [2,3]. A TCA is an antidepressant that binds to cholinergic, adrenergic, and histaminic receptors, and patients who are prescribed TCAs often experience unwanted side effects and intolerances, such as sedation, dizziness, reduced cognitive function, and increased heartrate [2,4]. TCA users are also at a high risk of potential drug interactions due to its highly protein-bound mechanism, the long half-life of metabolites (8–80 h), and the extensive metabolism by CYP450 enzymes [4].

Previous observational studies have reported the consistent association of the risk of falls or fall-related injuries with SSRI and SNRI users, which was often higher than that of TCA users [5,6,7,8,9]. A multinational cohort study by Tamblyn et al. (2020) also reported the association between fractures and SSRI use in several countries (i.e., Canada, the United Kingdom, and the United States) [7]. TCA use was associated with a significantly increased risk of fractures in the United States and Taiwan only, which was explained by higher doses of TCA being used in the United States and imipramine being the preferred drug choice in Taiwan [7].

While antidepressant dose and drug choice were investigated as contributing factors to the risks of each antidepressant subclass, the drug–drug interaction in older adults is another aspect that should be considered. A recent case crossover study of SSRI and SNRI users with the concomitant use of CYP2D6-inhibiting drugs reported a significant risk of fall-related injuries in adults aged 70 years or older [10]. In contrast, the risk of fall-related injuries was not elevated in TCA users with the concomitant use of CYP2D6-inhibiting drugs [10]. This case crossover study conveyed the possible effects of concurrent medications on the risk of fall-related injuries of different antidepressant subclasses.

Given the limited evidence on antidepressant use associated with fall-related injuries, this study was conducted (a) to investigate the effects of antidepressant use on the risk of fall-related injuries using national elderly cohort data and (b) to identify factors influencing the risk of fall-related injuries after classifying antidepressant drugs, polypharmacy, and central nervous system (CNS) drugs by therapeutic classes. This study aims to provide a more profound understanding of the antidepressant-associated risk of fall-related injuries concerning concurrent medications and to add to the limited existing evidence in Asian countries using a nationwide claims database in South Korea.

## 2. Materials and Methods

### 2.1. Study Design and Data Sources

**Figure 1:**
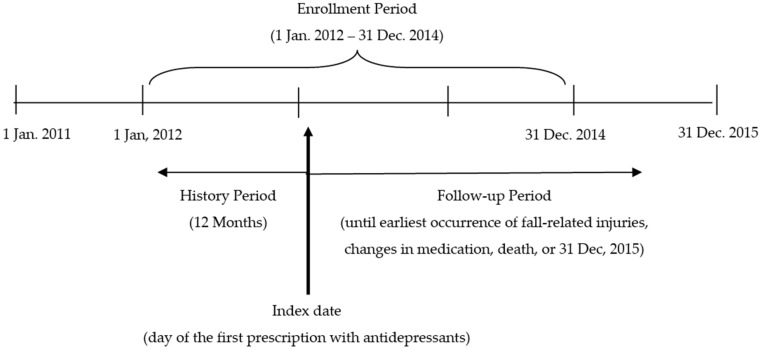
The study design for eligible patient selection.

A matched cohort study was conducted using the senior cohort dataset provided by the Korean national health insurance service (NHIS), which is a nationwide health insurer for all citizens in Korea. This study identified antidepressant users who were aged 70 to 89 years at the time of their first prescription antidepressant use during the enrollment period of between 1 January 2012 and 31 December 2014 (Figure 1). The date of their first antidepressant prescription was defined as the index date. This study included new antidepressant users who did not have a prescription for antidepressants for one year prior to the index date to minimize confounding bias due to use of antidepressants.

The eligible antidepressant users were followed from the index date until a treatment change concerning the antidepressant (i.e., discontinued, switched or augmented with another antidepressant), a fall-related injury, death or the end of 2015, whichever occurred earliest. The drug was considered discontinued if the subsequent prescription had a gap longer than the grace period, which was 200% of the prescribed duration of the previous prescription [11,12]. Non-antidepressant users were those patients who never used antidepressants during the entire study period and their index dates were randomly assigned during the study period. These patients were also followed from the index date until a fall-related injury, death or the end of 2015, whichever occurred earliest.

The NHIS senior cohort included 558,147 older adults, selected using stratified sampling method from 5.5 million adults of 60 years of age or older in Korea during 2002–2015 [13]. The NHIS senior database contained information about insurance eligibility (e.g., income decile and demographics), medical service provider information, medical claims data for disease diagnoses (based on the International Classification of Disease, 10th Revision (ICD-10) codes), services received, prescription drug records, and results of biennial national health examinations. This study was approved by the Institutional Review Board of the Chung-Ang University (1041078-201708-HRSB-162-01). Study reporting followed the Strengthening the Reporting of Observational Studies in Epidemiology (STROBE) guidelines.

### 2.2. Study Population

**Figure 2:**
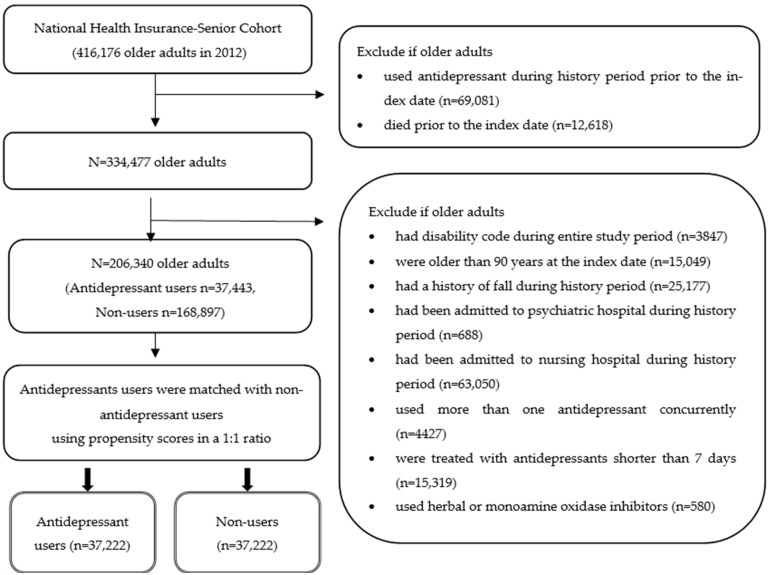
The process of study patient selection.

Study patients were further screened based on the following inclusion and exclusion criteria, as described in Figure 2. Patients were excluded if they were 90 or more years old at the index date, had a history of fall-related injuries during the history period prior to the index date or were admitted to a psychiatric or nursing hospital during the history period prior to the index date [14,15,16]. Patients were also excluded if they either used antidepressants for less than seven days or more than one antidepressant concurrently. These exclusion criteria were established to account for existing fall risks or potential reversal causality. The users of monoamine oxidase inhibitors and herbal classes were excluded due to the small sample size.

The eligible antidepressant users were matched to non-antidepressant users based on propensity scores. The matching by propensity scores was conducted based on the greedy method, without replacement by the propensity score, within caliper 0.1 widths to minimize the difference and balance covariates that could affect the outcome [17]. The propensity scores of antidepressant use were calculated using logistic regression with adjustment for income, living in a metropolitan area, the experience of hospitalizations (yes/no), the number of outpatient visits (0~7, ≥8), and various comorbidities (i.e., osteoarthritis/rheumatoid arthritis, malignant neoplasm, cardiovascular diseases, thyroid diseases, hypertension, osteoporosis, neurologic diseases, and gastric ulcers) [6,7,15]. A total of 37,222 antidepressant users were matched with the same number of non-antidepressant users.

### 2.3. Definition of Fall-Related Injuries

Fall-related injuries were a primary outcome of this study. An event was considered a fall-related injury if the ICD-10 diagnosis code of the accidental fall (W00-W19) was identified as a primary or secondary diagnosis. The following injury codes in primary diagnosis were also defined as fall-related injuries: (a) fall-related fractures of the skull and facial bones (S02, excluding S02.5 and S02.9), the neck (S12), the ribs, sternum, and thoracic spine (S22, excluding S22.5), the lumbar spine and pelvis (S32), the shoulder and upper arm (S42), the forearm (S52), the wrist and hand (S62), the femur (S72), the lower leg (S82), the calcaneus (S92.0), and multiple body regions (T02); (b) intracranial injury (S06); or (c) joint dislocations of the (jaw (S03.0), the shoulder (S43.0), the elbow (S53.0 and S53.1), the wrist (S63.0), the knee (S83.0 and S83.1), and multiple body regions (T03) [18,19]. Events involving transport accidents (V0–V99) or pathological/stress fractures (M84.3, M84.4, M48.4, and M90.7) were excluded to eliminate injuries from other causes.

In contrast to previous studies, the definition of fall-related injuries was not limited to the records of emergency department visits or hospitalizations because only about 56 percent of patients visit an outpatient clinic with fall-related injuries in Korea [20]. Instead, only the primary diagnosis of injury codes was assessed to increase specificity and capture fall-related injuries from the claims database.

### 2.4. Exposure to Medications

The primary exposure of interest was the use of antidepressants. The Anatomic Therapeutic Classification system (ATC), developed by the World Health Organization (WHO), was used to identify the antidepressant agents (N06A), which are presented in Supplementary Table S1 [21].

The antidepressants were further classified as tricyclic antidepressants (TCAs), selective serotonin reuptake inhibitors (SSRIs), serotonin norepinephrine reuptake inhibitors (SNRIs), selective serotonin reuptake enhancers (SSREs), and others, according to the Korean Pharmaceutical Information Center (KPIC) drug classification (Supplementary Table S1) [22]. The number of total medications and CNS classes prescribed with antidepressants was counted based on the drugs classified using ATC fifth level codes (Supplementary Table S2) and the CNS class, respectively, during the 30-day period prior to the fall-related injury outcome, changes in antidepressant use or 31 December 2015, whichever came earliest [23,24]. The CNS class included opioids (N02A), antiepileptics (N03), antipsychotics (N05A), anxiolytics (N05B), hypnotics and sedatives (N05C), and antidepressants (N06A). Polypharmacy was defined as having more than 10 medications (including antidepressants) prescribed concurrently [25].

### 2.5. Covariates: Demographics, Health Service Utilization, and Comorbidities

We assessed the characteristics of patient demographics (i.e., age, gender, living area, income) and comorbidities in the 365-day period prior to the index date and the health service utilization (i.e., hospitalization experience, number of outpatient visits) in the 90-day period prior to the index date.

The patients’ comorbidities were identified during the 12-month history period prior to the index date using the ICD-10 codes. Considering data availability and association with the risk of falls, this study included eight commonly identified comorbidities based on previous studies and the guideline for prevention of falls [5,6,7,15,26]: osteoarthritis/rheumatoid arthritis (M15–M19, M05–M06), malignant neoplasm (C00–26, C30–34, C37–41, C43, C45–58, C60–85, C88, C90–97), cardiovascular diseases (I20–I28, I30–I52 except I46), thyroid diseases (E00–E07), hypertension (I10–I15), osteoporosis (M80–M82), neurologic diseases (G20-G26, G40, G47, G60–G64, G80–G83, R25–R29), and gastric ulcers (K25–K28). These comorbidities were used in the model to adjust for the risk of fall-related injuries in the analysis.

### 2.6. Data Analyses

The differences in sociodemographic characteristics, health services utilization, and comorbidities between the antidepressant users and the matched non-users were compared using standardized difference, for which sample size was less of a factor, and differences of less than 0.1 were considered negligible [27,28,29].

After patients were stratified by the subclass of antidepressant, the usage patterns of all medications, central nervous system medications, and the daily dose of antidepressant were examined. Antidepressant doses were calculated by dividing the daily dose based on prescription claims (the total dose dispensed divided by the total number of days of supply) by the defined daily dose recommended by WHO [21].

The incidence rates of fall-related injury were calculated by dividing the number of fall-related injuries by 1000 person years. The Cox proportional hazard model was used to estimate the hazard ratio (HR) of fall-related injuries in each antidepressant class and for each agent, both with and without adjusting for age (70–79, 80–89), gender, comorbidities (psychiatric disease, anemia, chronic lung disease, diabetes mellitus, stroke/TIA, urinary incontinence, and sensory impairment), and the status of polypharmacy (0–10, >10 drugs) [25]. The risk of fall-related injuries was calculated for each class of antidepressant (TCAs, SSRIs, SSREs, and SNRIs). In addition, the subgroup analyses examined the risk of TCA, SSRI, SSRE, SNRI, and Trazodone users stratified by the number of total medications (0–9, ≥10) and by the number of CNS classes (0,1, ≥2) prescribed concurrently. All statistical analyses were conducted using SAS version 9.3 (SAS Institute Inc., Cary, NC, USA).

## 3. Results

A total of 74,444 older adults were included for analyses after applying the exclusion criteria and the matching (Figure 2). The mean age of users and non-users was 76.7 years with a standard deviation (SD) of 4.5 (Table 1). Most users and non-users were female (61.0%). More than half of the study population had osteoarthritis/rheumatoid arthritis and hypertension. All demographic variables, health service utilization, and comorbidities were balanced between the antidepressant users and the non-users (standardized difference < 0.1).

The mean number of total medications prescribed concurrently ranged from 11.9 (SSRI user) to 13.0 (SNRI/Trazodone user), and the mean number of CNS class medications ranged from 1.8 (SSRE user) to 2.3 (Trazodone user) (Table 2). Approximately half of the antidepressant users were prescribed more than 10 medications, which is considered polypharmacy. The mean number of prescribed medications was similar among the antidepressant users, but the prescribed categories of the total medications and the CNS classes differed. The nine most frequently used therapeutic drug classes (except for the CNS class) that were potentially associated with fall-related injuries are listed in Table 2. Anti-inflammatory and antirheumatic agents were prescribed for more than half of TCA, SSRE, and SNRI users. Opioids were prescribed most frequently for TCA and SNRI users, and anxiolytics for SSRI and Trazodone users. The mean dose of antidepressant in each subclass was as low as 0.15 DDD (Trazodone user) and as high as 0.82 DDD (SSRI user). TCAs were prescribed in very low doses (mean DDD 0.25, SD 0.16), which is because they are often prescribed in low doses for conditions other than depression [30].

All antidepressant subclasses were associated with a higher risk of fall-related injuries compared to non-users, except for the SSRE subclass (Table 3). The risk was highest in SNRI users (adj-HR = 1.54, 95% CI = 1.21–1.92), followed by other antidepressant users (adj-HR = 1.47, 95% CI = 1.16–1.86), SSRI users (adj-HR = 1.42, 95% CI = 1.19–1.71), and TCA users (adj-HR = 1.21, 95% CI = 1.03–1.42). Among SSRIs, paroxetine (adj-HR = 1.71, 95% CI = 1.18–2.49) had the highest risk of fall-related injuries, but sertraline and fluoxetine did not significantly increase the risk. Patients prescribed duloxetine (adj-HR = 1.59, 95% CI = 1.24–2.03), imipramine (adj-HR = 1.50, 95% CI = 1.07–2.11), and trazodone (adj-HR = 1.51, 95% CI = 1.17–1.96) had the highest risk of fall-related injuries among the SNRI, TCA, and other antidepressant classes, respectively.

**Figure 3:**
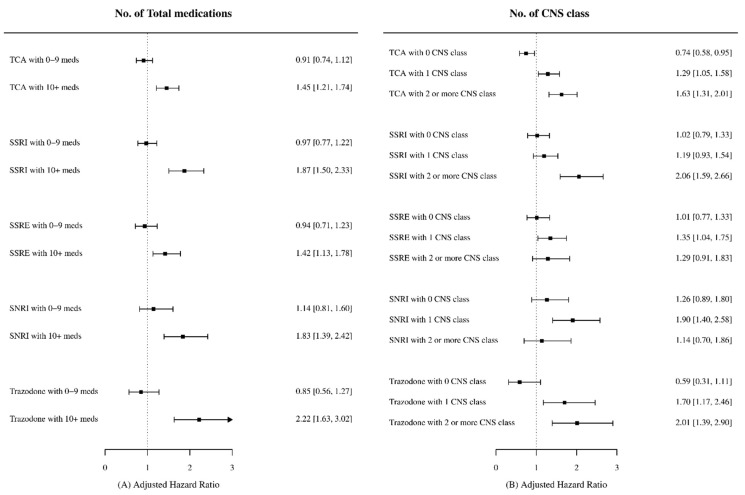
The subgroup analyses of antidepressant classes by (**A**) the number of total medications and (**B**) the CNS class associated with fall-related injuries.

The risk of fall-related injuries for patients using antidepressants was significantly increased by polypharmacy (>10 medications) and CNS class medications (Figure 3). The risk of fall-related injuries of all therapeutic classes of antidepressant was increased with an adjusted hazard ratio of 1.42–2.22 when patients were currently treated with more than 10 medications. The risk of fall-related injuries was not significantly increased when CNS class medication was not used concurrently. However, the risk was significantly increased when antidepressants were used concurrently with CNS class medications. The magnitude of risk was different depending on therapeutic class of antidepressant and the number of CNS class medications. SSRI users had a significantly increased risk of fall-related injuries when using ten or more total medications (adj-HR = 1.87, 95% CI = 1.50–2.33) and two or more CNS class medications (adj-HR = 2.06, 95% CI = 1.59–2.66). This significant risk of fall-related injuries was observed in SNRI users with polypharmacy (adj-HR = 1.83, 95% CI = 1.39–2.42) including one CNS class medications (adj-HR = 1.90, 95% CI = 1.40–2.58). TCA users had an increased risk of fall-related injuries with polypharmacy (adj-HR = 1.45, 95% CI = 1.21–1.74) including one and two or more CNS class medications (adj-HR = 1.29, 95% CI = 1.05–1.58; HR = 1.63, 95% CI = 1.31–2.01).

## 4. Discussion

In this study, the risk of fall-related injuries for users of SNRIs, other antidepressants, SSRIs, and TCAs increased by 54, 47, 42, and 21 percent, respectively. Consistent with previous studies, SSRI users had a higher risk of fall-related injuries than TCA users [6,8]. Paroxetine, trazodone, and imipramine users had particularly high risks, which correlated well with their known pharmacological properties: paroxetine has anticholinergic and antihistaminergic activity linked with more sedation compared to other SSRIs; imipramine is associated with a higher incidence of orthostatic hypotension, which can contribute to a higher risk of fall-related injuries; and trazodone is known for dizziness and sedation by blocking alpha1 adrenergic and histaminergic receptors [2,4,31].

The less preferred use of the high-risk agent imipramine and the lower dose of TCAs used in Korea may have contributed to the lower fall risk of the TCA subclass. This conclusion is also consistent with previous multinational studies reporting a lower risk of fractures for TCAs users in the countries with no preferred use of imipramine [7]. TCA users with less than 10 total medications or no other CNS class medications also had either reduced or statistically not significant risks of fall-related injuries. This implies that TCAs alone may have minimal effects on the risk of fall-related injuries and contribute to the lower risk of TCA users (adj-HR = 1.21 95% CI = 1.03–1.42). Another discovery of this study was that tianeptine, categorized as an SSRE and mainly used in East Asian countries, was not associated with fall-related injuries.

While all antidepressant classes were not significantly associated with fall-related injuries when no other CNS class medication was prescribed, there was a twofold increase in the risk of fall-related injuries for TCA, SSRI, and Trazodone users when the antidepressants were used concurrently with two or more CNS medications. These results are comparable to the cohort study of the Australian veteran population using three or more CNS drugs (incidence rate ratio = 1.96, 95% CI = 1.48–2.43) [32]. A study of three or more standardized daily doses (the daily dose divided by the minimum effective geriatric daily dose) of CNS related medications being used by nursing home residents also reported a twofold increase in the risk of serious injurious falls (adjusted OR = 1.83, 95% CI = 1.35–2.48) [31]. Based on the previous studies mentioned above, the risk of fall-related injuries with a CNS drug alone was not as significant as that of combined use with multiple CNS classes. Similarly, the risk of fall-related injuries with antidepressant medications could mainly result from antidepressants being used concurrently with other CNS class medications, rather than antidepressant use alone. Further studies are needed to explore the risk of CNS classes when being used without antidepressants. TCA and Trazodone users showed a significantly increased risk with a lower threshold of CNS class (one or more) medications compared to SSRI users (two or more). No association between SNRI and SSRE users with two or more CNS class medications and fall-related injuries was found in this study, which may be the result of the relatively low-dose use of SNRIs and the smaller number of CNS class medications prescribed in those subclasses.

The CNS class medications may have additional effects to the side effects of TCAs, such as cognitive impairment and sedation, contributing to a significantly increased risk of fall-related injuries with concurrent use. The higher number of total medications and CNS class medications yielded a greater risk for those using SSRIs. The large number of concurrent medications in SSRI users may double the fall-related injury risk by the potential drug–drug interactions with the SSRIs. The results from previous case crossover studies relate to the results of this study by reporting the increased risk of fall-related injuries in SSRI users with concomitant use of CYP2D6-inhibiting drugs and the non-significant association with TCA users in the older population (70 years or older) [10]. The effects of the number of concurrent medications on SSRI users compared to TCA users may be a plausible explanation for the previous observational reporting of the greater risk of falls or fractures for TCA users. Such potential risks could be the result of less frequent drug–drug interactions with TCAs due to the low-dose prescribing. Further studies in this area are recommended for future analyses.

The associations between the risk of falls and drugs with strong anticholinergic effects or those inducing orthostatic hypotension were found to be controversial [33,34,35,36,37]. A recent systematic review concluded that the risk of falls is higher in patients with a moderate–high use of anticholinergics (i.e., the use of more than one anticholinergic), but this risk is less clear for patients with low usage (i.e., the use of only one anticholinergic) [33]. Antihypertensives, diuretics, and anti-Parkinson drugs that were potentially causing orthostatic hypotension were reported to have no association with falls in the previous meta-analysis [34,35]. Loop diuretics have been shown to be associated with falls [34]. However, one study found that the association was dependent on the duration of loop diuretics use: the risk was significant in patients taking loop diuretics for longer than two years, but was not significant for shorter periods of use (adjusted OR = 1.38, 95% CI = 0.94–2.03) [36]. Further studies are recommended to investigate the association between these medications and falls in the future.

A strength of this study is the use of a large database to perform a population-based study. The cohort was representative of older adults in Korea and allowed us to have detailed information about medication use. Possible confounders, such as smoking, depression severity, and functional status, were not able to be obtained by the claims database; however, to minimize the potential for residual confounding, this study attempted to adjust for demographics, health service utilization, comorbidities, and the number of concurrent medications by propensity matching and regression. the initial therapy of antidepressants was also considered as an exposure to include patients with similar conditions and to avoid prevalent user bias. The study was not limited to depression symptoms because antidepressants were used more frequently for other symptoms in older Korean adults. To the best of our knowledge, this was the first study to explore the risk of fall-related injuries by the subclasses of antidepressants with the stratification of the number of concurrent medications (total medications and CNS class medications). Such an analysis was possible using real-world evidence only, and it has provided practical information on the safe use of antidepressants alongside other drugs in older adults. However, this study did not examine the exposure period nor the dose of concurrent medications, which is recommended for future studies.

In addition, since the outcome of the study was based on the operational definition of ICD-10 codes used in previous studies, misclassification bias could occur. This study attempted to minimize the bias and adapt the definition appropriately to the Korean population. The categories of injury and ICD-10 codes were based on the definitions of serious fall injuries and fall-related injuries provided in previous studies and mapped appropriately to the ICD-10 codes [18,19]. The diagnostic codes associated with fall-related injuries highly correlated with a Korean survey study of falls that reported different injuries associated with the falls [20].

Antidepressant pharmacotherapy is essential for treating depression and preventing suicide among older adults. Older Korean adults were reported having the highest suicide rate among OECD countries from 2011 to 2017 [38]. Moreover, antidepressant use has been expanding to include use in smoking cessation, diabetic neuropathy, fibromyalgia, irritable bowel syndrome, postherpetic neuralgia, and other conditions [39,40]. Antidepressants are recommended instead of opioids for older adults for pain-related symptoms [41]. Given the expanding use of antidepressants in older adults, appropriate drug use should be thoroughly investigated within the context of fall prevention.

## 5. Conclusions

This study concluded that the risk of fall-related injuries was significantly increased by the use of antidepressants, including duloxetine, escitalopram, paroxetine, amitriptyline, imipramine, and trazodone, in older Korean adults. The risk of fall-related injuries for patients using antidepressants was significantly increased when the patients were treated with polypharmacy or CNS class medications.

When antidepressants are prescribed to older adults, healthcare providers should carefully consider factors including the dose, the number of concurrently administered medications and the therapeutic classes of CNS. It is also recommended that unnecessary medications are de-prescribed to minimize potential adverse drug reactions in older adults. This study suggests further investigation on potential drug–drug interactions with antidepressants that may result in a higher risk of fall-related injuries.

## Figures and Tables

**Table 1:** The baseline characteristics of the antidepressant users and the matched non-users.

	Antidepressant Users (*n* = 37,222)	Matched Non-Users (*n* = 37,222)	Standardized Difference
	N (%)	N (%)	
Demographics			
Age, mean (±SD)	76.7 (±4.5)	76.7 (±4.5)	
70–79	27,733 (74.5)	27,770 (74.6)	0.00
80–89	9489 (25.5)	9452 (25.4)	
Income			
0–2nd quintile	11,722 (31.5)	11,517 (30.9)	0.01
3–4th quintile	10,499 (28.2)	10,447 (28.1)	0.00
5–10th quintile	15,001 (40.3)	15,258 (41.0)	0.01
Female	22,691 (61.0)	22,691 (61.0)	0.00
Metropolitan ^a^	13,925 (37.4)	13,811 (37.1)	0.01
Health Service Utilization			
Hospitalization ^b^	4554 (12.2)	4297 (11.5)	0.02
No. of outpatient visits ^b^			
0–7	16,508 (44.4)	16,183 (43.5)	0.02
≥8	20,714 (55.6)	21,039 (56.5)	
**Comorbidities**			
OA/RA	19,920 (53.5)	20,070 (53.9)	0.01
Malignant neoplasm	3157 (8.5)	3042 (8.2)	0.01
Cardiovascular diseases	9760 (26.2)	9739 (26.2)	0.00
Thyroid diseases	5240 (14.1)	4901 (13.2)	0.03
Hypertension	25,514 (68.5)	25,607 (68.8)	0.01
Osteoporosis	10,588 (28.4)	10,440 (28.0)	0.01
Neurologic diseases	9459 (25.4)	9213 (24.8)	0.02
Gastric ulcers	13,150 (35.3)	13,192 (35.4)	0.00

SD: standard deviation; OA: osteoarthritis; RA: rheumatoid arthritis; ^a^ Living in metropolitan area; ^b^ 90-day period before the index date.

**Table 2:** The usage patterns of the medications used concurrently with the antidepressants.

Antidepressant Users	TCAs (*n* = 16,127) N (%)	SSRIs (*n* = 5621) N (%)	SSREs (*n* = 9401) N (%)	SNRIs (*n* = 3146) N (%)	Trazodone (*n* = 2290) N (%)
No. of total medications, mean (±SD)	12.5 (±7.7)	11.9 (±8.7)	12.6 (±7.4)	13.0 (±8.0)	13.0 (±10.1)
0–10	7634 (47.3)	3079 (54.8)	4329 (46.1)	1445 (45.9)	1167 (51.0)
>10	8493 (52.7)	2542 (45.2)	5072 (54.0)	1701 (54.1)	1123 (49.0)
No. of CNS medications, mean (±SD)	2.1 (±0.9)	2.1 (±0.9)	1.8 (±0.8)	1.9 (±0.9)	2.3 (±1.0)
0	4885 (30.3)	1661 (29.6)	3825 (40.7)	1195 (38.0)	513 (22.4)
1	6592 (40.9)	2328 (41.4)	3743 (39.8)	1252 (39.8)	920 (40.2)
≥2	4650 (28.8)	1632 (29.0)	1833 (19.5)	699 (22.2)	857 (37.4)
Therapeutic classes of medications (except for CNS classes)					
Anti-inflammatories and antirheumatics	9146 (56.7)	1772 (31.5)	4788 (50.9)	2167 (68.9)	847 (37.0)
Calcium channel blockers	6415 (39.8)	2172 (38.6)	3567 (37.9)	1274 (40.5)	943 (41.2)
Antithrombotic agents	6464 (40.1)	2530 (45.0)	3499 (37.2)	1335 (42.4)	1013 (44.2)
Angiotensin II antagonists	2703 (16.8)	999 (17.8)	1553 (16.5)	570 (18.1)	438 (19.1)
H2-receptor antagonists	7086 (43.9)	1852 (33.0)	4467 (47.5)	1423 (45.2)	769 (33.6)
Lipid modifying agents	4653 (28.9)	1818 (32.3)	2592 (27.6)	1049 (33.3)	662 (28.9)
Drugs used for diabetes	3515 (21.8)	1199 (21.3)	1753 (18.7)	833 (26.5)	498 (21.8)
Cough and cold preparations	2871 (17.8)	1128 (20.1)	1780 (18.9)	583 (18.5)	553 (24.2)
Antihistamines for systemic use	3186 (19.8)	912 (16.2)	1750 (18.6)	522 (16.6)	471 (20.6)
CNS medications					
Opioids	6445 (40.0)	1121 (19.9)	3089 (32.9)	1350 (42.9)	512 (22.4)
Antiepileptics	3152 (19.5)	593 (10.6)	684 (7.3)	418 (13.3)	272 (11.9)
Antipsychotics	413 (2.6)	614 (10.9)	126 (1.3)	74 (2.4)	317 (13.8)
Anxiolytics	5651 (35.0)	2919 (51.9)	3085 (32.8)	732 (23.3)	1137 (49.7)
Hypnotics and sedatives	1474 (9.1)	846 (15.1)	803 (8.5)	219 (7.0)	721 (31.5)
DDD prescribed, mean (±SD)	0.25 (±0.16)	0.82 (±0.37)	0.75 (±0.24)	0.57 (±0.22)	0.15 (±0.09)
>0–≤0.5 DDD	14,974 (92.9)	2049 (36.5)	1513 (16.1)	2430 (77.2)	2278 (99.5)
>0.5–≤1.0 DDD	1125 (7.0)	3019 (53.7)	7844 (83.4)	681 (21.7)	12 (0.5)
>1.0 DDD	28 (0.2)	553 (9.8)	44 (0.5)	35 (1.1)	0 (0)

CNS: central nervous system; SNRIs: serotonin norepinephrine reuptake inhibitors; SSREs: selective serotonin reuptake enhancers; SSRIs: selective serotonin reuptake inhibitors; TCAs: tricyclic antidepressants; DDD: defined daily dose.

**Table 3:** The incidence rates and risks of fall-related injuries according to antidepressant.

	Fall-Related Injuries (FRIs)					
	No. of Patients	Person Years (PYs)	No. of FRIs	Incidence/ 1000 PYs *	Crude HR (95% CI)	Adjusted HR (95% CI) **
Age						
70–79	55,503	12,546.0	976	77.8	Reference	Reference
80–89	18,941	4722.9	490	103.8	1.41 (1.25–1.58)	1.36 (1.21–1.53)
Female	45,382	10,367.6	998	96.3	1.35 (1.20–1.52)	1.37 (1.19–1.58)
**Antidepressants**						
All	37,222	8797.5	978	111.2	1.25 (1.09–1.44)	1.30 (1.13–1.50)
**By Class of Antidepressant**						
SNRIs						
All	3146	759.9	102	134.2	1.48 (1.18–1.87)	1.54 (1.21–1.92)
Duloxetine	2623	606.3	86	141.8	1.55 (1.21–1.98)	1.59 (1.24–2.03)
Milnacipran	351	106.8	11	103	1.19 (0.65–2.19)	1.23 (0.67–2.25)
SSREs						
All	9401	1582.6	175	110.6	1.19 (0.98–1.45)	1.20 (0.99–1.46)
Tianeptine	9401	1582.6	175	110.6	1.19 (0.98–1.45)	1.20 (0.99–1.46)
SSRIs						
All	5621	2050.1	234	114.1	1.34 (1.12–1.60)	1.42 (1.19–1.71)
Escitalopram	3573	1397.6	160	114.5	1.35 (1.11–1.65)	1.43 (1.17–1.75)
Sertraline	657	228.1	26	114	1.33 (0.89–1.99)	1.44 (0.96–2.17)
Paroxetine	643	225.1	31	137.7	1.62 (1.12–2.36)	1.71 (1.18–2.49)
Fluoxetine	583	129.5	11	84.9	0.95 (0.52–1.74)	0.99 (0.54–1.81)
TCAs						
All	5621	2050.1	234	114.1	1.15 (0.98–1.36)	1.21 (1.03–1.42)
Amitriptyline	3573	1397.6	160	114.5	1.19 (1.00–1.42)	1.23 (1.03–1.46)
Nortriptyline	657	228.1	26	114	0.98 (0.75–1.29)	1.03 (0.78–1.35)
Imipramine	643	225.1	31	137.7	1.27 (0.91–1.77)	1.50 (1.07–2.11)
Other						
All	2927	788.3	96	121.8	1.39 (1.10–1.77)	1.47 (1.16–1.86)
Trazodone	2290	589.6	75	127.2	1.45 (1.12–1.88)	1.51 (1.17–1.96)
Mirtazapine	443	123.2	16	129.8	1.49 (0.90–2.46)	1.58 (0.95–2.62)

PY: person years; HR: hazard ratio; CI: confidence Interval; SNRIs: serotonin norepinephrine reuptake inhibitors; SSREs: selective serotonin reuptake enhancers; SSRIs: selective serotonin reuptake inhibitors; TCAs: tricyclic antidepressants; * antidepressant agents with more than 100 person years are presented in this table; ** hazard ratios were adjusted for age (70–79, 80–89), gender, comorbidities (psychiatric diseases, anemia, chronic lung disease, diabetes mellitus, stroke/TIA, urinary incontinence, and sensory impairment), and the status of polypharmacy (0–10, >10 drugs).

## Data Availability

The datasets used and analyzed in this study are available from the corresponding author on reasonable request.

## Supplementary Materials

The following supporting information can be downloaded at: https://www.mdpi.com/article/10.3390/ijerph19042298/s1, Table S1: Antidepressants classified by the Korean Pharmaceutical Information Center and defined daily dose (DDD) classified by WHO and Table S2: Classification of drugs using the Anatomical Therapeutic Chemical (ATC) classification code.

## References

[1] Kovich H., Dejong A. (2015). Common questions about the pharmacologic managmenet of depression in adults. Am. Fam. Physician.

[2] Dipiro J., Gary C., Poset L., Haines S., Nolin T., Ellingrod V. (2020). Pharmacotherapy: A Pathophysiologic Approach.

[3] Wang H., Bahk W., Seo J., Woo Y., Park Y., Jeong J., Kim W., Shim S., Lee J., Jon D. (2017). Korean Medication Algorithm for Depressive Disorder: Comparisons with Other Treatment Guidelines. Clin. Psychopharmacol. Neurosci..

[4] Hilal-Dandan R., Brunton L. (2014). Goodman and Gilman’s Manual of Pharmacology and Therapeutics.

[5] Marcum Z.A., Perera S., Thorpe J.M., Switzer G.E., Castle N.G., Strotmeyer E.S., Simonsick E.M., Ayonayon H.N., Philips C.L. (2016). Antidepressant use and recurrent falls in community-dwelling older adults: Findings from the health ABC study. Ann. Pharm..

[6] Coupland C., Dhiman P., Morriss R., Arthur A., Barton G., Hippisley-Cox J. (2011). Antidepressant use and risk of adverse outcomes in older people: Population based cohort study. BMJ.

[7] Tamblyn R., Bates D., Buckeridge D., Dixon W., Girard N., Haas J., Habib B., Iqbal U., Li J., Sheppard T. (2020). Multinational investigation of fracture risk with antidepressant use by class, drug, and indication. J. Am. Geriatr. Soc..

[8] Marci J., Iaboni A., Kirham J., Maxwell C., Gill S., Vasudev A., Whitehead M., Seitz D. (2017). Association between Antidepressants and Fall-Related Injuries among Long-Term Care Residents. Am. J. Geriatr. Psychatiry.

[9] Gagne J., Patrick A., Mogun H., Solomon D. (2011). Antidepressants and Fracture Risk in Older Adults: A Comparative Safety Analysis. Clin. Pharmacol. Ther..

[10] Dahl M., Leander K., Vikstrom M., Frumerie C., Nordenmalm S., Moller J., Soderberg-Lofdal K. (2021). CYP2D6 inhibiting drugs and risk of fall injuries after newly initiated antidepressant and antipsychotic therapy in a Swedish, register-based case-crossover study. Sci. Rep..

[11] Parker M., Moffet H., Adams A., Karter A. (2015). An algorithm to identify medication nonpersistence using electronic pharmacy databases. J. Am. Med. Inform. Assoc..

[12] Gardarsdottier H., Souverein P., Egbert T., Heerdink E. (2010). Construction of drug treatment episodes from drug-dispensing histories is influenced by the gap length. J. Clin. Epidemiol..

[13] Kim Y.I., Kim Y.Y., Yoon J.L., Won C.W., Ha S.J., Cho K.D., Park B.R., Bae S.J., Lee E.J. (2019). Cohort profile: National health insurance service-senior (NHIS-senior) cohort in Korea. BMJ Open.

[14] Ferrer A., Formiga F., Plana-Ripoll O., Tobella M., Gil A., Pujol R. (2012). Risk of falls in 85-year-olds is associated with functional and cognitive status: The Octabaix study. Arch. Gerontol. Geriatr..

[15] Moncada L.V.V., Mire L.G. (2017). Preventing falls in older persons. Am. Fam. Physician.

[16] Tinetti M.E., Speechley M. (1989). Prevention of falls among the elderly. N. Engl. J. Med..

[17] Austin P. (2011). Optimal caliper widths for propensity-score matching when estimating differences in means and differences in proportions in observational studies. Pharm. Statist..

[18] Kim S.B., Zingmond D.S., Keeler E.B., Jennings L.A., Wenger N.S., Reuben D.B., Ganz D.A. (2016). Development of an algorithm to identify fall-related injuries and costs in Medicare data. Inj. Epidemiol..

[19] Tinetti M., Han L., Lee D.S., McAvay G.J., Peduzzi P., Gross C.P., Zhou B., Lin H. (2014). Antihypertensive medications and serious fall injuries in a nationally representative sample of older adults. JAMA Intern. Med..

[20] Lee Y.G., Kim S.C., Chang M.S., Nam E.W., Kim S.G., Cho S.I. (2018). Complications and socioeconomic costs associated with falls in the elderly population. Ann. Rehabil. Med..

[21] WHO Collaborating Centre for Drug Statistics Methodology ATC Classification Index with DDDs. https://www.whocc.no/atc/structure_and_principles/.

[22] Korea Pharmaceutical Information Center KPIC Therapeutic Classification. https://www.health.kr/searchIngredient/KPICeffect.asp.

[23] American Geriatric Society Beers Criteria Update Expert Panel (2019). American Geriatric Society 2019 updated AGS Beers criteria for potentially inappropriate medications use in older adults. J. Am. Geriatr. Soc..

[24] Aspinall S., Springer S., Zhao X., Cunningham F., Thrope C., Semla T., Shorr R., Hanlon J. (2019). Central Nervous System Medication Burden and Risk of Recurrent Serious Falls and Hip Fractures in Veterans Affairs Nursing Home Residents. JAGS.

[25] Kim H.A., Shin J.Y., Park B.J. (2014). Prevalence and predictors of polypharmacy among Korean elderly. PLoS ONE.

[26] American Geriatrics Society, British Geriatrics Society, American Academy of Orthopaedic Surgeons Panel on Falls Prevention (2001). Guideline for the prevention of falls in older persons. J. Am. Geriatr. Soc..

[27] Mamdani M., Sykora K., Li P., Normand S.L.T., Streiner D.L., Austin P.C., Rochon P.A., Anderson G.M. (2005). Reader’s guide to critical appraisal of cohort studies: 2. Assessing potential for confounding. BMJ.

[28] Austin P. (2009). Balance diagnostics for comparing the distribution of baseline covariates between treatment groups in propensity-score matched samples. Statist. Med..

[29] Zhuo M., Hawley C.E., Paik J.M., Bessette L.G., Wexler D.J., Kim D.H., Tong A.Y., Kim S.C., Patorno E. (2021). Association of Sodium-Glucose Cotransporter–2 Inhibitors with Fracture Risk in Older Adults with Type 2 Diabetes. JAMA Netw. Open.

[30] Hwang J., Song I., Lee E., Ha D., Shin J. (2018). Prevalence and predidctors of tricyclic antidepressant use among elderly Koreans in primary-care and specialty clincs. Int. J. Clin. Pharmacol. Ther..

[31] Hanlon J., Zhao X., Naples J., Aspinall S., Perera S., Nace D., Castle N., Greenspan S., Thrope C. (2017). Central Nervous System Medication Burden and Serious Falls in Older Nursing Home Residents. JAGS.

[32] Pratt N., Ramsay E., Ellet L., Nguyen T., Barrat J., Roughead E. (2014). Association between Use of Multiple Psychoactive Medicines and Hospitalization for Falls: Retrospective Analysis of a Large Healthcare Claim Database. Drug Saf..

[33] Stewart C., Taylor-Rowan M., Soiza R.L., Quinn T.J., Loke Y.K., Myint P.K. (2021). Anticholinergic burden measures and older people’s falls risk: A systematic prognostic review. Ther. Adv. Drug Saf..

[34] de Vries M., Seppala L.J., Daams J.G., van de Glind E.M.M., Masud T., van der Velde N., EUGM Task and Finish Group on Fall-Risk-Increasing Drugs (2018). Fall-risk-increasing drugs: A systematic review and meta-analysis: I. cardiovascular drugs. J. Am. Med. Dir. Assoc..

[35] Figueroa J.J., Basford J.R., Low P.A. (2010). Preventing and treating orthostatic hypotension: As easy as A, B, C. Clevel. Clin. J. Med..

[36] Marcum Z.A., Perera S., Newman A.B., Thorpe J.M., Switzer G.E., Gray S.L., Simonsick E.M., Shorr R.I. (2015). Antihypertensive use and recurrent falls in community-dwelling older adults: Findings from the health ABC study. J. Gerontol. A Biol. Sci. Med. Sci..

[37] Seppala L.J., Wermelink A.M.A.T., de Vries M., Ploegmakers K.J., van de Glind E.M.M., Daams J.G., van der Velde N., EUGMS Task and Finish Group on Fall-Risk-Increasing Drugs (2018). Fall-risk-increasing drugs: A systematic review and meta-analysis: II. psychotropics. J. Am. Med. Dir. Assoc..

[38] Ministry of Health & Welfare—Korea Suicide Prevention Center (2020). Suicide Prevention Wite Book.

[39] Health Insurance Review &Assessment Service (2017). Detailed Criteria and Methods for Reimbursement of Medicaal Service (Drugs) and Its Evaluation Guidelines (Yoyang Geugyeo Jekyong Kijoon and Bangbub Sebu Sahang).

[40] Wong J., Montulsky A., Eaguale T., Buckeridge D., Brahamowicz M., Tamblyn R. (2016). Treatment Indications for Antidepressants Prescribed in Primary Care in Quebec, Canada, 2006–2015. JAMA.

[41] Park S., Kim W., Jang T. (2020). Opioids and Antidepressants for Pain Control in Musculoskeletal Disease. J. Korean Orthop. Assoc..

