# Supplementary material for: Risk of Fall-Related Injuries Associated with Antidepressant Use in Elderly Patients: A Nationwide Matched Cohort Study
Source: Int J Environ Res Public Health. 2022 Feb 17;19(4):2298. doi: 10.3390/ijerph19042298 (PMC8872471; doi:10.3390/ijerph19042298)
Supplement: Supplementary file 1 [file ijerph-19-02298-s001.zip › ijerph-1553646-supplementary.pdf]

Supplementary Table S1. Antidepressants classified by Korean Pharmaceutical Information Center and defined daily dose (DDD) by WHO

Subclasses/Drugs	ATC code	DDD
Antidepressant	N06A	
Tricyclic antidepressant (TCA)		
Amoxapine	N06AA17	150 mg
Imipramine	N06AA02	100 mg
Clomipramine	N06AA04	100 mg
Amitriptyline	N06AA09	75 mg
Nortriptyline	N06AA10	75 mg
Quinupramine	N06AA23	.
Selective serotonin reuptake inhibitor (SSRI)		
Fluoxetine	N06AB03	20 mg
Citalopram	N06AB04	20 mg
Paroxetine	N06AB05	20 mg
Sertraline	N06AB06	50 mg
Fluvoxamine	N06AB08	100 mg
Escitalopram	N06AB10	10 mg
Vortioxetine	N06AX26	10 mg
Serotonin norepinephrine reuptake inhibitor (SNRI)		
Venlafaxine	N06AX16	100 mg
Milnacipran	N06AX17	100 mg
Duloxetine	N06AX21	60 mg
Desvenlafaxine	N06AX23	50 mg
MAOI		
Moclobemide	N06AG02	300 mg
Selective serotonin reuptake enhancer (SSRE)		
Tianeptine	N06AX14	37.5 mg
Herbal		
St. John's Worth	N06AX25	.
Others		
Doxepin	N06AA12	100 mg
Trazodone	N06AX05	300 mg
Mirtazapine	N06AX11	30 mg
Bupropion	N06AX12	300 mg
Agomelatine	N06AX22	25 mg

Supplementary Table S2. Classification of drugs using Anatomic Therapeutic Chemical (ATC) classification code

Therapeutic drug class	ATC codes
Antacids	A02A
H2-receptor antagonists	A02BA
Proton pump inhibitors	A02BC-A02BD, M01AE52
Antispasmodics	A03A-A03E, A02AG, N02AG,A06AB3
Drugs for constipation	A06A
Drugs used in diabetes	A10
Antithrombotic agents	B01, C07FX02-C07FX04, C10BX01, C10BX02, C10BX04-C10BX06, C10BX08, C10BX12
Cardiac glycosides	C01A
Vasodilators	C02D, C04, C07E
Diuretics	C03, C07B- C07D, C08G,C09BA, C09DA, C09BX01, C09BX03, C09DX01, C09DX03, C10BX13
Beta blocking agents	C07, C09BX02
Calcium channel blockers	C08, C07FB, C09BB, C09DB, C09BX01, C09BX3, C09DX01, C09DX03, C10BX03, C10BX07, C10BX09, C10BX11, C10BX14
ACE inhibitors	C09A-C09B, C10BX13, C10BX04, C10BX06, C10BX07, C10BX11, C10BX12, C10BX14
Angiotensin II antagonists	C09C-C09D, C10BX10
Lipid modifying agents	C10, A10BH51
Urological	G04B
Drugs used in benign prostatic hypertrophy	G04C
Corticosteroids for systemic use	H02A
Anti-inflammatory/antirheumatic agents, non-steroid	M01A, N02AJ08, N02AJ14, N02AJ19
Muscle relaxants	M03B
Other analgesics, non-opioid	N02B, N02AJ01, N02AJ02, N02AJ03, N02AJ06, N02AJ07, N02AJ09, N02AJ13, N02AJ15, N02AJ17, N02AJ18
Antiparkinson drugs	N04
Antidementia drug	N06D
Drugs for obstructive airway disease	R03
Cough and cold preparations	R05
Antihistamines for systemic use	R06
